# High density DNA data storage library via dehydration with digital microfluidic retrieval

**DOI:** 10.1038/s41467-019-09517-y

**Published:** 2019-04-12

**Authors:** Sharon Newman, Ashley P. Stephenson, Max Willsey, Bichlien H. Nguyen, Christopher N. Takahashi, Karin Strauss, Luis Ceze

**Affiliations:** 1 School of Computer Science and Engineering, University of Washington, Seattle, WA 98195 USA; 2 Electrical Engineering Department, Stanford University, Stanford, CA 94305 USA; 3 Microsoft, Seattle, WA 98052 USA

## Abstract

DNA promises to be a high density data storage medium, but physical storage poses a challenge. To store large amounts of data, pools must be physically isolated so they can share the same addressing scheme. We propose the storage of dehydrated DNA spots on glass as an approach for scalable DNA data storage. The dried spots can then be retrieved by a water droplet using a digital microfluidic device. Here we show that this storage schema works with varying spot organization, spotted masses of DNA, and droplet retrieval dwell times. In all cases, the majority of the DNA was retrieved and successfully sequenced. We demonstrate that the spots can be densely arranged on a microfluidic device without significant contamination of the retrieval. We also demonstrate that 1 TB of data could be stored in a single spot of DNA and successfully retrieved using this method.

## Abstract (web summary)

DNA as a high density storage medium is receiving increasing attention, but long term physical storage is an unsolved problem. Here the authors show that up to 1 TB of data stored as dehydrated DNA spots on a glass cartridge can be retrieved in a spot of water using digital microfluidics with minimal data loss and contamination.

## Introduction

DNA is considered to be an attractive alternative medium for data storage, as it offers extreme information density (theoretically up to exabytes in a mm^3^), durability, and eternal format relevance^1–7^. Advancements in this area have primarily focused on synthesis, encoding/decoding, random access protocols, and the impact of emerging sequencing and synthesis technologies. However, the actual physical storage of DNA samples remains largely unexplored in the context of building DNA storage systems.

DNA data storage systems encode data into nucleotide sequences, which are chemically synthesized into oligonucleotide pools. While the theoretical information density of DNA is incredibly high, DNA data storage systems are practically constrained by the desire for random access, which allows the retrieval of specific data without sequencing the entire pool. Prior work has tagged oligonucleotide sequences with an unique address or identifier. Larger addresses use more nucleotides in the strand, but allow for more data to be stored in the pool. To retrieve a file, PCR and bead wash can effectively amplify the file of interest and filter out all oligonucleotides not associated with that file’s identifier, leaving only those of interest to be sequenced.

**Fig. 1:**
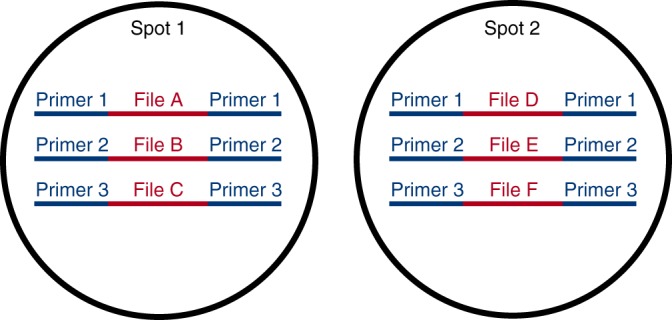
Files in physically isolated DNA spots can share the same addresses (primers). Physical isolation is necessary to scale beyond a single address space. The address of a spot would become part of a file’s address; in this example, file D would have address (Spot 2, Primer 1)

Random access is critical to the practicality of DNA storage systems, but the need for addressing each data item limits the capacity of a single pool to terabytes of information^7^. Physically isolated pools can use the same address space (Fig. 1), so further scaling requires physical isolation of pools and a system to organize and select them for data retrieval. DNA data must be in liquid form for sample preparation and sequencing. However, isolating liquid samples can be cumbersome, and the need for separate vessels sacrifices information density.

**Fig. 2:**
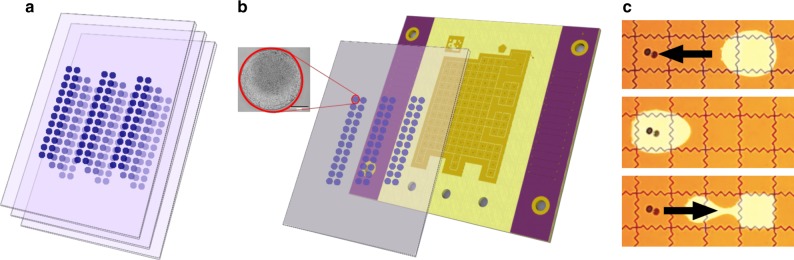
Storing physically isolated spots of dehydrated DNA on glass cartridges enables high information density. **a** Spotted cartridges can be further organized into decks. **b** To retrieve data, the desired cartridge is first loaded onto the DMF device consisting of an electrode grid used to perform retrieval functions. An actual magnified spot is depicted, where the scale bar in the inset is 275 um. **c** Next, a water droplet sandwiched between the cartridge and electrodes is actuated to move under the spotted DNA for rehydration. Depicted is an actual droplet moving on the device. After recovery, we manually recover the sample and analyzed it to assess DNA recovery rate and contamination across samples sharing paths on the DMF device

We present a DNA data storage architecture composed of many dehydrated DNA spots on a glass cartridge, shown in Fig. 2. The spots are physically isolated and individually retrievable using digital microfluidics (DMF) without contamination. The cartridges could be further organized in a deck and accessed using a multidimensional addressing system, like other scalable storage solutions such as tape or hard drives. Individual cartridges could store up to 50 TB of data using today’s DNA storage techniques. We report successful storage, retrieval, and decoding of DNA files of various sizes using this system, and we propose methods for library composition and organization.

Our solution is powered by a digital microfluidic device, a versatile class of fluidic automation systems that move individual droplets around on an array of electrodes via the electrowetting-on-dieletric phenomenon. Droplets are “sandwiched” between the electrode array and a common counter-electrode. Modulating an electric field between individual electrodes in the grid changes the interfacial surface tension of droplets, deforming them and causing them to move across electrodes^8^. The electrodes can be controlled individually, so DMF devices can be reprogrammed to take different actions without hardware changes.

DMF devices have been used for the manipulation of DNA, particularly for next generation sequencing (NGS) preparation^9–16^. Additionally, the use of dried reagents on a DMF device has been presented^17^. Our work differs as we intend to use the DMF device not for sample preparation but for storage and organization. We chose a DMF device over other fluid handling systems such as channel-based devices and pipetting robots due to the flexibility, relative low cost, and small footprint of DMF devices.

We evaluate the potential of our system by dehydrating spots of DNA, retrieving them with water droplets on the DMF device, and finally recovering, quantifying, and sequencing the results. Our results show that the droplets retrieves the majority of the DNA with low contamination from neighboring spots, making the technique suitable for DNA data storage.

## Results

### Evaluation of retrieval efficiency

To assess DNA retrieval efficiency, we conducted a series of experiments in which we stored and retrieved DNA using our DMF device. We varied spot mass, retrieval dwell time, and DNA file size.

In each experiment, we measured the mass of DNA retrieved relative to the DNA spotted. We also recorded sequencing metrics of coverage and unread sequences (the percentage of unique sequences present in the file that were not found in sequencing data).

To evaluate the effects of spot mass on retrieval efficiency, we tested DNA spots with masses ranging from 5 ng to around 60 ng, where spot mass was determined by multiplying the spot volume prior to dehydration by the DNA sample solution concentration. Each spot consisted of multiple copies of 2042 unique sequences (roughly 20 KB of data), so the copy number scales with the mass. We retrieved on average 79% of the spotted DNA mass after 60 s of dwell time. Aggregating all trials, over 99.8% of the sequences were read at least once. We observed that, regardless of the initial spot mass, enough DNA was recovered to successfully decode with no bit errors.

In addition, we performed retrieval with various dwell times (i.e., the time droplets remain under the DNA spot, rehydrating its contents) ranging from 1 to 120 s. Each spot consisted of multiple copies of 2042 unique DNA sequences with a mass of roughly 60 ng. While dwell time is correlated with the mass retrieved, we still retrieve a significant fraction (77%) of DNA mass with just a 1 s dwell time. Sequencing and subsequent file decoding was successful on all tests and was uncorrelated with dwell time. We also successfully stored and retrieved larger files in DNA spots. In these tests, we stored 398 k copies of 276,000 unique sequences (roughly 2.7 MB) in spots of roughly 30 ng. We recovered 94% of the DNA by mass, and successfully sequenced the file with only 1.2% of the unique sequences missing.

Finally, we demonstrated the ability to retrieve three different files consecutively with a single water droplet. The droplet moved onto each DNA spot in succession, and the product was sequenced. All three files (indexed separately) were successfully recovered with only 0.5–1.4% of unique sequences missing.

### Evaluation of contamination

To explore the feasibility of densely storing many DNA spots on a single DMF cartridge, we tested retrieval within configurations of several spotted DNA files to determine how contamination affects file recovery. We investigated three potential contamination sources: oil, neighboring spot, and path contamination.

**Fig. 3:**
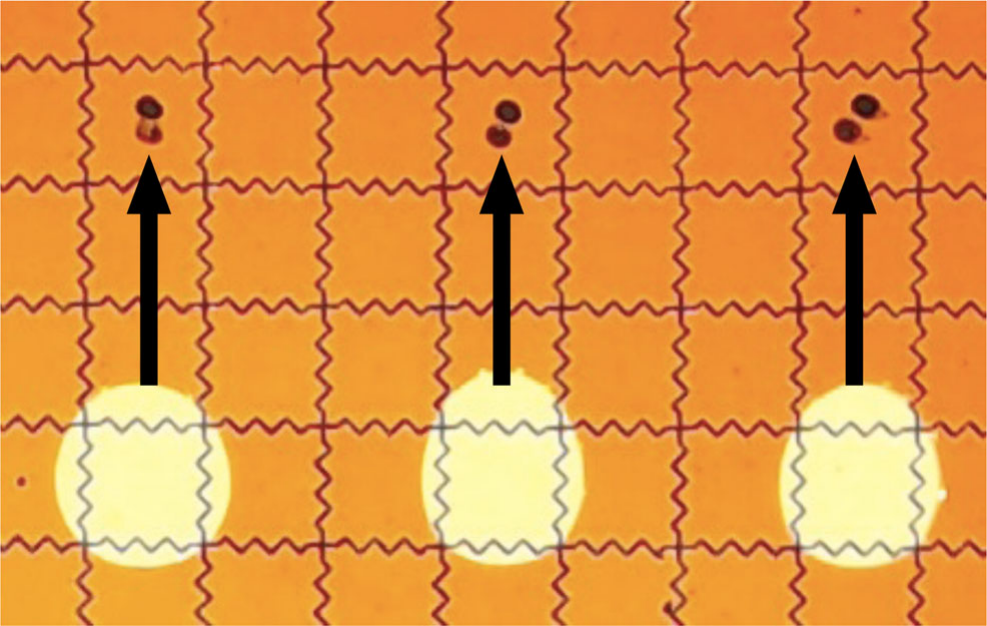
DMF setup to test for contamination through the oil. Black ink is used to visually mark DNA spot locations, and light circular blobs are water droplets. Droplets moved in tandem up to the spot, stayed for 60 s, and returned to their original location

To test for contamination through the oil, we performed three retrievals at the same time (Fig. 3) and tested for contamination. We moved the three droplets to spots above them at the same time, let them dwell for 60 s, and sequenced the left-most droplets. Table 1 shows the sequencing results for that droplet. The rows show that very little of the other files was picked up from the left-most droplet.

**Table 1:** Contamination results

Experiment type	Mass spotted (ng)	% recovered	Coverage	% with 0 reads	
				Actual	Expected
Oil contamination: left	65	74	33.26	0.10	0
Oil contamination: middle	65	99	0.01	99.07	100
Oil contamination: right	69	46	0	100.00	100
Neighbor contamination: NW	65	−	0.02	98.24	100
Neighbor contamination: N	65	−	0.02	98.04	100
Neighbor contamination: NE	69	−	0.10	91.14	100
Neighbor contamination: W	72	−	0.15	87.02	100
Neighbor contamination: center	63	71	617.60	0	0
Neighbor contamination: E	60	−	0.02	98.43	100
Neighbor contamination: SW	49	−	0.07	93.39	100
Neighbor contamination: S	68	−	0.05	95.00	100
Neighbor contamination: SE	61	−	0.02	98.33	100
Path contamination 1	72	105	0	100.00	100
Path contamination 2	63	103	0	100.00	100
Path contamination 3	60	92	43.45	0.05	0

We also show the expected % with 0 reads with the ideal of no contamination. Oil contamination**:** Only the left droplet was sequenced; sequencing data in all rows represents presence of that file in the left droplet. Neighbor contamination**:** The sequenced droplet visited the center spot; sequencing data in all rows represents presence of that file in the sequenced droplet. Path contamination**:** Numbers 1–3 indicate the droplets as indicated in Fig. 5a–d. Only droplet 3 was sequenced; sequencing data in all rows represents presence of that file in droplet 3. Droplet 3 was analyzed by NGS for existence of droplets 1–3

**Fig. 4:**
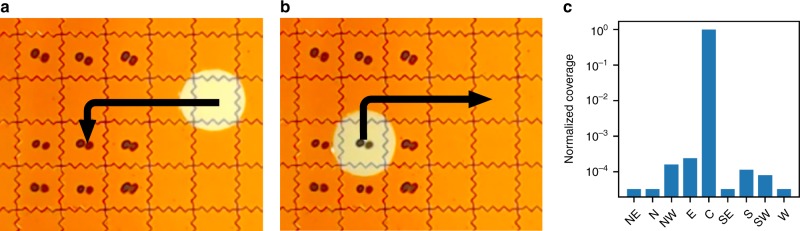
DMF setup for neighbor contamination tests. **a** Nine uniquely indexed pools are spotted onto a 4 x 3 electrode array with a central row left as an empty path for the recovery droplet. Cardinal directions of file locations are in reference to the central target spot. A droplet is moved to the central storage pool. **b** The droplet with reconstituted file returns to starting point for manual retrieval and analysis. **c** NGS coverage of each discovered file in cardinal coordinates (log scale) as seen in Table 1

To test for contamination from densely arranged neighboring spots, we tested retrieval in a 3 × 4 array of spots separated by a row of empty spots (Fig. 4). The droplet moved into the row, stayed on the center spot for 60 s, and then moved back out. Table 1 shows that the droplet retrieved very little of the neighboring spots.

**Fig. 5:**
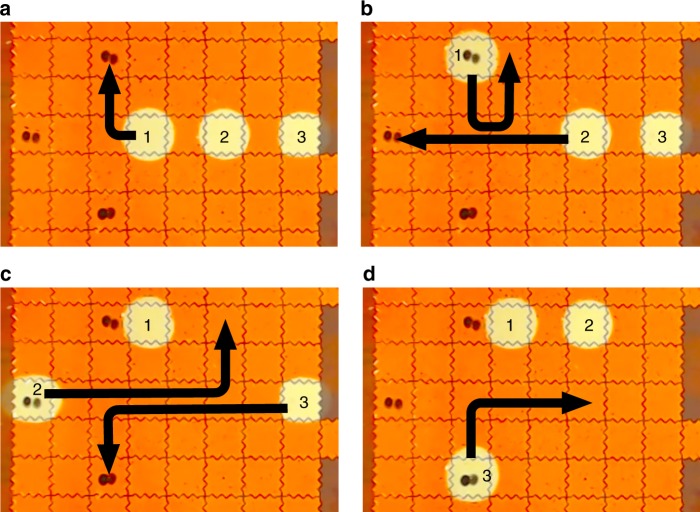
DMF setup for path contamination tests. **a** Three different files are spotted at different locations on the cartridge. Three retrieval droplets are initiated on the DMF device. Droplet 1 is moved to the north-most file. **b** Droplet 1 moves to its final coordinate. Droplet 2 then moves to the west-most file, passing two electrodes Droplet 1 had moved across. **c** Droplet 2 moves to its final coordinate. Droplet 3 moves to the south-most file, using a part of the path both droplets 1 and 2 had used. **d** Droplet 3 moves to its final coordinate for manual retrieval and analysis

We next tested the role of path contamination in file recovery by spotting different DNA files on a cartridge and moving water droplets along the same path during consecutive retrievals of each file (Fig. 5). We only sequenced the last droplet (droplet 3) to do the retrieval, and we found no evidence of file contamination along pathways (Table 1, Path Contamination).

### Scaling to a terabyte per spot

To demonstrate 1 TB of data per spot, we needed to perform an experiment with more DNA than those we initially performed. Our 315-base double stranded DNA has a 110-base data payload region, with additional regions necessary for ligation and sequencing (see Supplementary Fig. 1). However, if synthesized directly, 175 bases would be usable for data, due to no longer needing primers for ligation. The 315-base strand has a molecular weight^18^ of 1.9 × 10^5^ g mol^−1^. Previous work^7^ has demonstrated a data density of 8.1 × 10^−1^ bits/base, or 1.0 × 10^−1^ bytes/base. From these figures, the following calculation demonstrates that 18 ng (successfully sequenced) will contain 1 TB of data.

1 $$\frac{{1.0 \times 10^{12}\,{\mathrm{bytes}}}}{{{\mathrm{spot}}}} \times\frac{{{\mathrm{base}}}}{{1.0 \times 10^{ - 1}\,{\mathrm{bytes}}}} \times \frac{{{\mathrm{strand}}}}{{175\,{\mathrm{bases}}}} \\ \times \frac{{{\mathrm{mol}}}}{{6.0 \times 10^{23}{\mathrm{strand}}}} \times \frac{{1.9 \times 10^5{\mathrm{g}}}}{{{\mathrm{mol}}}} = \frac{{1.8 \times 10^{ - 8}{\mathrm{g}}}}{{{\mathrm{spot}}}}$$

However, not all of the DNA in a spot would be successfully sequenced. Some may not be retrieved at all, and some duplication is needed to deal with sequencing errors. So to demonstrate retrieval of 1 TB of data, we allowed for 2× loss in spot retrieval (consistent with results from Table 2) and an additional 5× for sequencing duplication^7^. So we proceeded to spot and retrieve in excess of 2 × 5 × 18 ng = 180 ng to demonstrate that 1 TB can be retrieved from a single spot. Table 3 shows that retrieval and subsequent quantification was in line with the other results, demonstrating that 1 TB of data could be successfully stored and retrieved from a spot.

**Table 2:** Retrieval results

# Unique sequences	Dwell time (s)	Spot mass (ng)	% recovered	Coverage	% sequences w/ 0 reads
File Size					
**2042**^a^	—	—	81.6 ± 20	19.7 ± 18.35	1.02 ± 2.6
** 276,000**	60	33	94	74.3	1.19
Mass tests					
2042	60	**5**	54	22.17	0.05
2042	60	**13**	94	18.96	0.1
2042	60	**19**	67	23.69	0.15
2042	60	**42**	88	18.45	0.08
2042	60	**63**	80	12.20	0.13
Timing tests					
2042	**120**	65	102	36.6	0.835
2042	**60**	58	83	14.3	0.1
2042	**30**	64	87	32.6	0.33
2042	**5**	70	82	9.9	3.98
2042	**1**	50	77	34.0	0.05
Multiple file retrieval with single droplet					
2042	60	54	—	8.04	1.4
2042	60	61	—	10.13	0.47
2042	60	60	—	9.93	1.14

The bold text emphasizes the quantities varied in a set of experiments

^a^This row does not represent a single experiment but the average of all other experimental runs with the 2042 unique sequences file. Numbers after the ± are standard deviation

**Table 3:** Retrieval results for larger spot masses (greater than 180 ng, see Section 2) to demonstrate feasibility of storing 1 TB of data in a single spot

# Unique sequences	Dwell time (s)	Spot mass (ng)	% recovered
2042	120	201	66
2042	120	210	56
2042	120	209	55

## Discussion

DNA-based data storage is currently impractical due to the high cost and slow speed of synthesis and sequencing. However, these costs are expected to decrease rapidly^19^. As this technology approaches feasibility, more research on fluidics automation and sample storage will be necessary to ensure these components do not limit the applications of DNA-based data storage.

We presented a physical DNA storage solution where dehydrated spots of DNA are stored on glass plates on a digital microfluidic device. Our design is amenable to automation since rehydration and retrieval is performed on the DMF device. Therefore, this work is complementary to the use of DMF devices for DNA sample prep^9–16^.

Multiple spots can be arranged on a single glass plate, further increasing the storage density. On our 127 electrode DMF device, 50 electrodes could comfortably fit in alternating rows (similar to Fig. 4), leading to 50 TB of data per glass plate.

This work further suggests a hierarchical storage scheme. Many glass cartridges could be stacked compactly into decks as shown in Fig. 2a, only moving onto the DMF device for retrieval. Furthermore, the DNA stored each individual spot could utilize addressing schemes designed for random access, allowing for retrieval of specific files from the sample.

We picture future work leading to a three tier storage scheme with three different retrieval mechanisms. Cartridges could be retrieved from decks by a robotic device, similar to how tape and disk drives are stored in data centers today. Spots can be retrieved from cartridges by digital microfluidics as shown in this work. Finally, specific files can retrieved from spots by PCR-based random access^7^. Files in different spots could share the same address space (see Fig. 1). Such a system could realize the oft-hyped data density of DNA-based data storage.

## Methods

### Glass cartridge preparation

An indium tin oxide coated glass plate (Delta Technologies CB-90IN-S211) was spin-coated (Laurell Technologies WS-650Mz-23NPPB) in house with a hydrophobic layer of Teflon AF 1600 (98 × *g* for 60 s, DuPont/Chemours). Two microliter volumes of a ligated DNA sample solution (diluted as necessary to achieve various DNA spot masses by volume) were dropped onto the glass plate (Delta Technologies) at marked locations and dried in an oven at 40 °C for 30 min

### DMF device construction

We constructed a custom made DMF device based on the OpenDrop platform^20^. The device consists of a printed circuit board (PCBway) patterned with an array of 127 2.7 mm square electrodes, depicted in Supplementary Fig. 2. The PCB electrode arrays were sputtered with a 5-μm dielectric layer of Parylene C by the Washington Nanofabrication Facility and subsequently spin-coated with a layer of Teflon AF 1600 as described for the glass plates. The glass cartridge containing the spotted files served as the top plate in our two-plate DMF device, which was assembled as shown in Supplementary Fig. 3. A custom electronic interface was used to automate the actuation (130–200 Vrms, 490 Hz AC square waveform) of individual electrodes and control the movement of water droplets on the electrode array.

### Spot retrieval

A 4-μL (2%/0.04 μL precision^21^) droplet of molecular biology grade water was first dispensed onto an unoccupied electrode. Three-hundred-micrometer thick electrical tape spacers were placed at the perimeter of the PCB to accommodate fluids between the electrode array and the top plate. The top plate was placed over the spacers and silicone oil (1–5cst, Sigma–Aldrich) was added between the plates to facilitate droplet movement and prevent droplet evaporation. The water droplet was then moved to the electrode location corresponding to the file of interest and allowed to dwell for a specified time to rehydrate the DNA spot. Depending on which experiment was performed, the droplet paths and dwell times varied. For the retrieval efficiency experiments, nine uniquely indexed pools of DNA were spotted onto the cartridge. The spots were arranged in a 4 × 3 electrode array with a central row left as an empty path for the retrieval droplet. For each retrieval, a single water droplet was initiated on the DMF device, moved to its target file, allowed to sit for 1–120 s for file reconstitution, and then returned to its starting location. For the contamination evaluation experiments, three different files were spotted at different locations on the cartridge. Three retrieval water droplets were initiated on the DMF device, and then moved along a common path to their respective file locations in successive order as shown in Fig. 5. Droplet movements through the paths described were accurate and reproducible. This movement is demonstrated in Supplementary Movie 1.

Following each experiment, the top plate was removed and the droplet(s) were collected manually with a pipette for quantification.

### DNA quantification

DNA concentrations were measured using either a ThermoFisher Nanodrop or Qubit 4 Fluorometer. When necessary, concentrations were measured using qPCR (primer sequences available in data package). Multiple concentration measurements were taken for each sample and averaged to calculate the percentage mass recovered values reported.

### DNA preparation workflow

DNA strands were designed as shown in Supplementary Fig. 1.

DNA files were synthesized by Twist Biosciences. A primer with a random 25-nucleotide overhang was used to enrich the files during PCR amplification. After purification using a QIAquick PCR purification kit, the files were split into aliquots. Each aliquot was prepared for sequencing using the Illumina TruSeq Nano kit and tagged with an unique Illumina index. Ligated samples were purified using Illumina sample purification beads and enriched by PCR. Successful ligation was confirmed using a QIAxcel Advanced system.

Note: the addition of the random 25N regions helps promote diversity during the clustering cycles (4–7 sequencing cycles) in the NextSeq, and the separate indices allows all the samples to be sequenced in the same run^7,22^.

### Next-generation sequencing (NGS) workflow

After experiments on the DMF platform, concentrations of each sample droplet were measured, and the samples were mixed together in equal proportions. Then the mixed sample was prepared following the NextSeq System Denature and Dilute Libraries Guide with a spike-in of 10–20% of the PhiX genome, which is an Illumina sequencing control^7,23^.

### Image analysis

Images of dried DNA were captured using an Olympus BX53M upright optical microscope and analysis were performed using ImageJ software.

### DNA spot characterization

Spots of DNA were pipetted onto the glass cartridge in locations marked with a sharpie marker that corresponded to the underlying DMF device electrodes. Spots were centered on each of the neighboring 2.7 × 2.7 mm electrodes used to store the DNA library. Representative dehydrated DNA spots are shown in Supplementary Fig. 4.

We measured an average spot diameter of 1151 μm with a standard deviation of 483.5 μm. Variations in spot shape and size are likely the result of differing droplet impact velocities upon manually pipetting each DNA sample onto the cartridge for subsequent dehydration^24^. With a maximum measured diameter of 2030 μm, which is smaller than both the electrode and the retrieval droplet, we do not believe these variations had any significant effects on DNA reconstitution and file recovery.

## Supplementary information

Supplementary Information

Description of Additional Supplementary Files

Supplementary Movie 1

## Data Availability

All data are presented in the tables and figures of the paper. Sequencing data and primers can be found here: https://github.com/uwmisl/2019-spotted-dna-data. Any additional data are available from the authors.
