# Supplementary material for: High density DNA data storage library via dehydration with digital microfluidic retrieval
Source: Nat Commun. 2019 Apr 12;10:1706. doi: 10.1038/s41467-019-09517-y (PMC6461645; doi:10.1038/s41467-019-09517-y)
Supplement: Supplementary file 1 — Supplementary Information [file 41467_2019_9517_MOESM1_ESM.pdf]

High Density DNA Data Storage Library via Dehydration with Digital Microfluidic Retrieval

(Supplementary Information)

Newman et al.

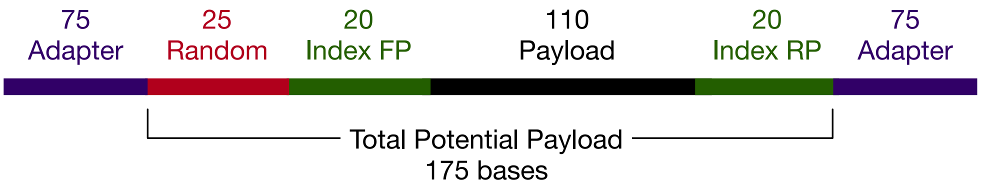

Supplementary Figure 1: DNA strand layout.

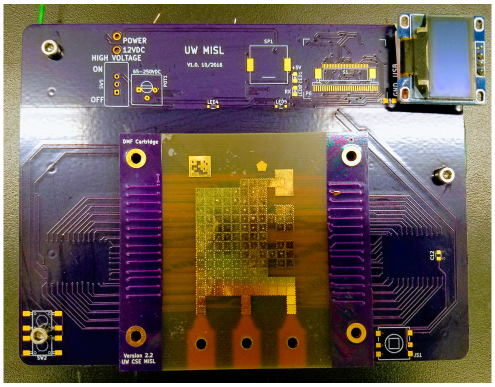

Supplementary Figure 2: The DMF system we built for this study. The purple PCB supports the electronic components needed to actuate the electrodes. The gold region contains the electrode grid on which water droplets travel for file retrieval. The DNA spotted cartridge (not shown) is placed on top of the electrode grid during operation such that the water droplets are in contact with both the electrodes and the cartridge surface, which serves as the counter-electrode.

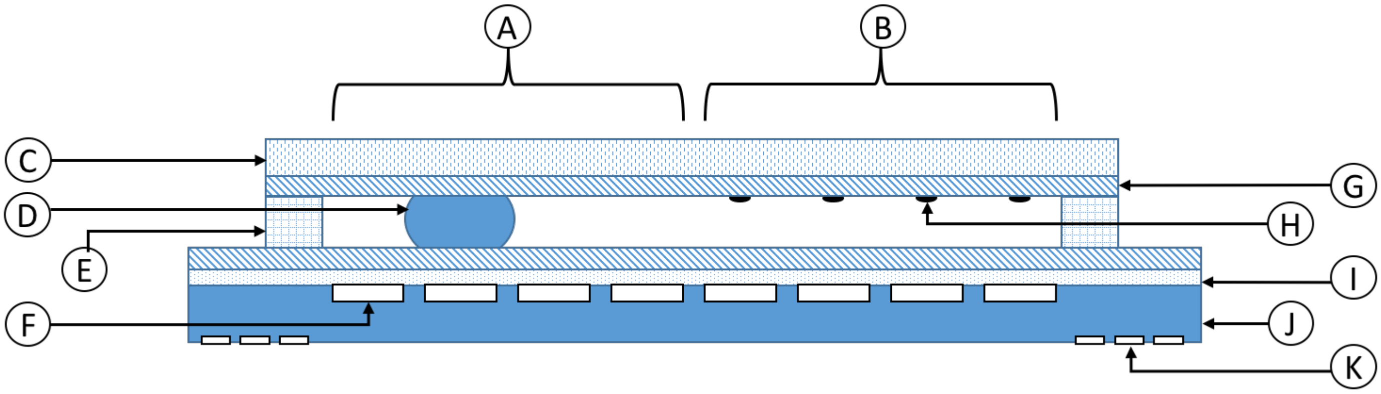

Supplementary Figure 3: A cross section of our DMF device. A: Working area where recovery droplets are initialized and where basic operations such as merging, mixing and splitting can take place. B: Pool storage area where dried spotted DNA (H) is placed adjacent to empty rows (not shown) for droplet access. C: Top conductive glass plate and library cartridge. D: Example actuated droplet. E: Mechanical spacers. F: Top metal PCB layer containing the active electrodes. G: Hydrophobic Teflon AF coating. H: Spotted DNA for long term storage. I: Parylene C dielectric layer. J: Modular PCB cartridge. Different electrode layouts may be used depending on needs. K: Electrical “pogo” pads used as the electrical interface between the PCB cartridge and control logic.

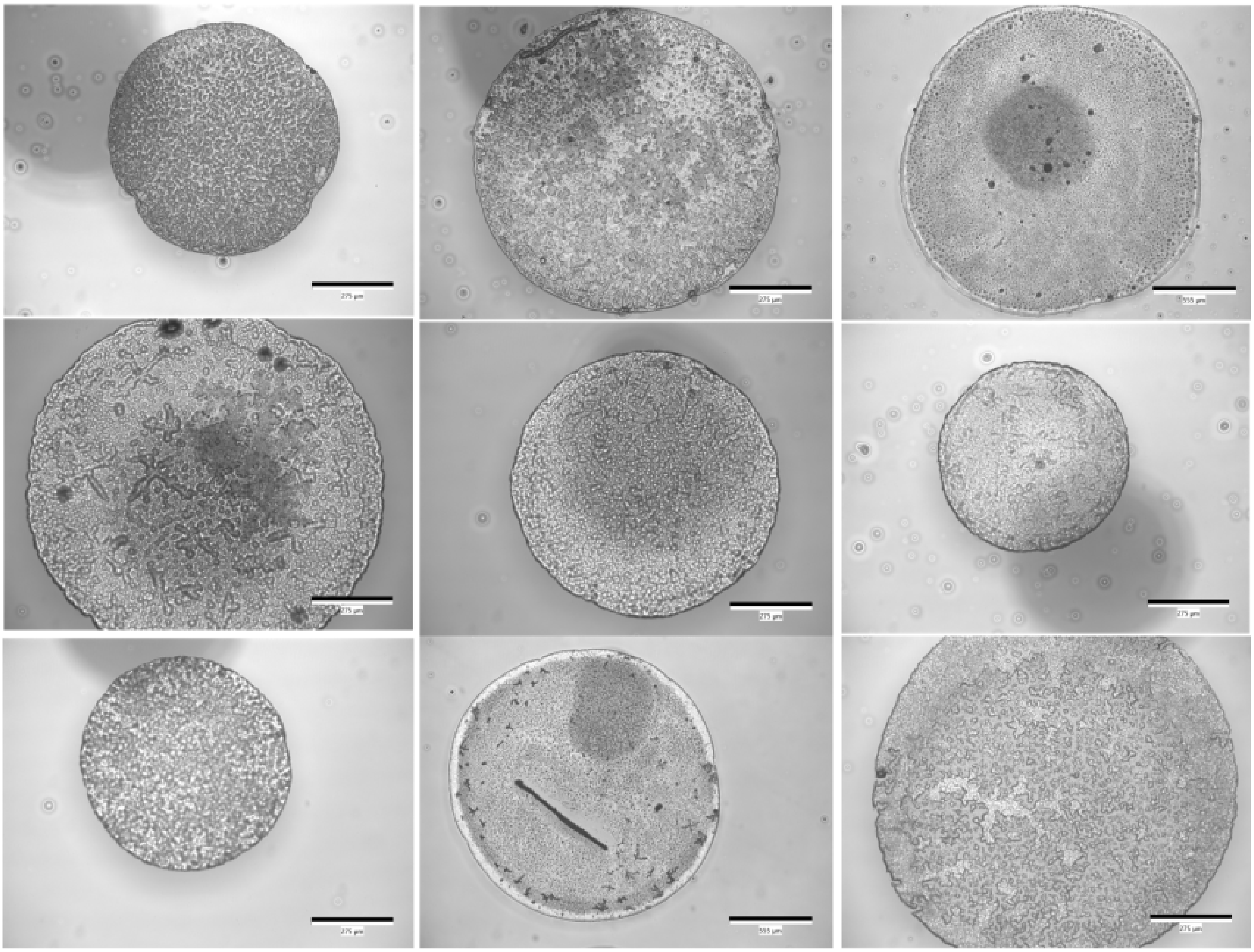

Supplementary Figure 4: Representative DNA spot images taken with a 5X objective after dehydration on the glass cartridge.
