# Supplementary material for: High density DNA data storage library via dehydration with digital microfluidic retrieval
Source: Nat Commun. 2019 Apr 12;10:1706. doi: 10.1038/s41467-019-09517-y (PMC6461645; doi:10.1038/s41467-019-09517-y)
Supplement: Supplementary file 2 — Description of Additional Supplementary Files [file 41467_2019_9517_MOESM2_ESM.docx]

**Title:** Supplementary Movie 1
**Description:** Representative droplet movement on our DMF device. Three retrieval droplets travel successively through a shared path to their respective file locations for DNA reconstitution.
